# Polymorphisms in the Egl nine homolog 3 (*EGLN3*) and Peroxisome proliferator activated receptor-alpha (*PPARα*) genes and their correlation with hypoxia adaptation in Tibetan chickens

**DOI:** 10.1371/journal.pone.0194156

**Published:** 2018-03-15

**Authors:** ChengLin Zhong, SiChen Li, JingJing Li, FengPeng Li, MingXia Ran, LingYun Qiu, DiYan Li, Qing Zhu, Yan Wang, HuaDong Yin, Gang Shu, Chaowu Yang, XiaoLing Zhao

**Affiliations:** 1 Farm Animal Genetic Resources Exploration and Innovation Key Laboratory of Sichuan Province, Sichuan Agricultural University, Chengdu, Sichuan Province, P. R. China; 2 Department of Pharmacy, College of Veterinary Medicine, Sichuan Agricultural University, Chengdu, Sichuan Province, P. R. China; 3 Sichuan Academy of Agricultural Sciences, Chengdu, Sichuan Province, P. R. China; 4 Department of Animal Science, College of Animal Science and Technology, Sichuan Agricultural University, P. R. China; Universita degli Studi di Bologna, ITALY

## Abstract

*Peroxisome proliferator activated receptor-alpha (PPARα)* and *Egl nine homolog 3* (*EGLN3)* play critical roles in facilitating the adaptation to a hypoxic environment. However, the relationship between *EGLN3* and *PPARα* variants and hypoxic adaptation remains poorly understood in Tibetan chickens. To better understand the effects of genetic variation, we sequenced exons of *PPARα* and *EGLN3* in 138 Lowland chickens (LC) from 7 breeds that were located in Emei, Miyi, Shimian, Wanyuan, Pengxian, and Muchuan in the Sichuan province, and Wenchang in the Hainan province (altitudes for these locations are below 1800 meters). Total 166 Tibetan chickens (TC) from 7 subpopulations that were located in Shigatse, Lhoka, Lhasa, Garze, Aba, Diqing and Yushu in the Tibetan area were also sequenced (altitudes greater than 2700 meters). One single-nucleotide polymorphism (rs316017491, C > T) was identified in *EGLN3* and was shared by TC and LC with no significant difference for allele frequencies between them (P > 0.05). Six single-nucleotide polymorphisms (SNP1, A29410G; SNP2, rs13886097; SNP3, T29467C; SNP4, rs735915170; SNP5, rs736599044; and SNP6, rs740077421) including one non-synonymous mutation (SNP2, T > C) were identified in *PPARα*. This is the first report of SNP1 and SNP3. There was a difference between TC and LC for allele frequencies (P <0.01), except for SNP1, SNP4, and SNP5) The fix index statistic test indicated that there was population differentiation between TC and LC for SNP2, SNP3, and SNP6 in *PPARα* (P < 0.05). Phylogenetic analysis showed that the genetic distance among chickens, finch and great tit were close for both *EGLN3* and *PPARα*. Bioinformatics analysis of *PPARα* showed that SNP2 leads to an amino acid substitution of Ile for Met, which results in the protein being more likely to be hydrolyzed. Thus, genetic variation in *PPARα* may play a role in the ability of TC to adapt to a high altitude environment; however we were unable to identify a relationship between polymorphisms in *EGLN3* and environmental adaptability.

## Introduction

Tibetan chickens, an aboriginal chicken breed distributed in the highland at over 3,000 m, have adapted to the harsh living conditions, characterized by cold weather, low partial pressure of oxygen and strong ultraviolet radiation [1]. Compared with the breeds that inhabit the Lowland, Tibetan chickens have more erythrocytes with enhanced oxygen affinity, richer blood vessel density and less mean corpuscular volume. All of these changes were produced by strong selection pressures during the history of domestication [2, 3], which may directly affect the genetic structure of this population.

Peroxisome proliferator-activated receptors (*PPARs*), as members of the nuclear hormone receptor superfamily, play a key role in energy metabolism [4]. The ability to consume oxygen and to produce adenosine triphosphate (ATP) during energy metabolism greatly influences the ability of animals to adapt to hypoxia [5]. There are three isotypes named *PPARα*, *PPARβ*, and *PPARγ*. *PPARα* is the main regulator of lipid metabolism [6]. Carbohydrate and lipid metabolism are two important components of energy metabolic pathways. Animals utilize one as an optimal-fuel strategy to cope with cold hypoxic environments [7]. Previous studies indicated that the genes undergoing positive selection in the ground tit on the Tibetan plateau were mostly involved in fatty-acid metabolic pathways [8]. Most animals use fatty acids as energetic substrate, as mitochondrial β-oxidation contributes to energy production via oxidative phosphorylation, thereby generating ATP [5, 9]. The activated-*PPARα* modulates this pathway by up-regulating the gene expression of some key factors such as fatty acid transporter protein (FATP), carnitine palmitoyl transferase I (CPT I), and acetyl-CoA synthetase (ACS) [10].

In addition to energy metabolic factors, the hypoxia-inducible factor-1α (*HIF-1α*) is vital to oxygen regulation. *Egl nine homolog 3* (*EGLN3)*, also called proline hydroxylase domain 3 (*PHD3*), controls the expression of the *HIF-1α* gene [11, 12]. When oxygen is present, *PHD3* hydroxylates specific proline residues on HIF-1α, initiated by von Hippel-Lindau protein (pVHL), leading to ubiquitination and destruction of the HIF-1α protein. In a hypoxic environment, the activity of *EGLN3* decreased, leading to accumulation of HIF-1α and formation of erythrocytes, which improved oxygen transportation [13].

We hypothesized that sequence variation in *PPARα* and *EGLN3* genes may contribute to the adaptation to hypoxic conditions in Tibetan chickens. Thus, we identified SNPs in the coding sequences of each gene in Tibetan chicken (TC) and Lowland chicken (LC)s and examined their association.

## Materials and methods

### Sampling and DNA extraction

**Fig 1:**
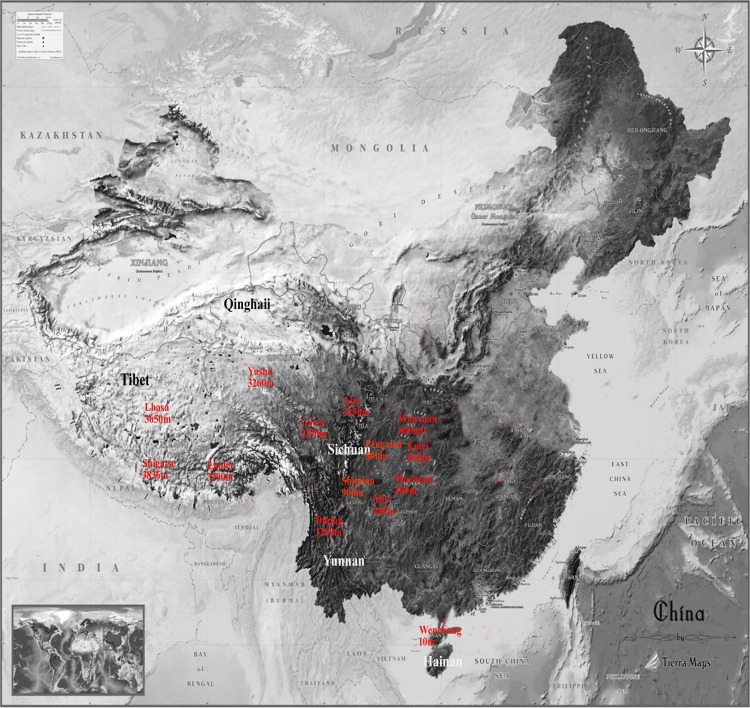
The locations of nine chicken populations. The black and white font represents the five provinces in which all populations in this study are distributed. The red font represents the sampling location of the experimental material. Shigatse, Lhoka, Lhasa, Garze, Aba, Diqing, and Yushu are the main areas where Tibetan chickens are distributed. Emei, Miyi, Shimian, Wanyuan, Pengxian, Muchuan, and Wenchang are the main areas where Lowland chickens are distributed.

In total, 304 blood samples were collected from 7 highland locations in Qinghai, Tibet, and Yunnan, and the Sichuan province, including Shigatse, Lhoka, Lhasa, Garze, Aba, Diqing, and Yushu, and 7 lowland native chicken breeds in Emei, Miyi, Shimian, Wanyuan, Pengxian, and Muchuan in the Sichuan province and Wenchang in the Hainan province (Fig 1). Blood was collected from the brachial vein and genomic DNA was extracted via the phenol-chloroform method [14]. The altitude, longitude, latitude, and population size of each location are shown in Table 1.

**Table 1:** Altitude, longitude, and latitude of the sampling locations for 7 Tibetan subpopulations and 7 Lowland chicken breeds.

Populations	Sampling locations	Sample sizes	Altitude (m)	Longitude (E)	Latitude (N)
**Tibetan chicken (TC)**					
Shigatse (RKZ)	Shigatse, Tibet	11	3900	89.60	28.92
Lhoka (SN)	Lhoka, Tibet	24	3700	90.03	28.27
Lhasa (LS)	Lhasa, Tibet	28	3650	91.01	29.26
Garze (GZ)	Garze, Sichuan	7	3390	99.22	28.34
Aba (AB)	Aba, Sichuan	25	3300	102.33	31.27
Diqing (DQ)	Diqing, Yunnan	19	3280	99.53	28.08
Yushu (YS)	Yushu, Qinghai	52	2700	96.6	33.2
**Lowland chicken (LC)**					
Emei (EM)	Emei, Sichuan	9	1800	103.41	29.49
Miyi (MY)	Panzhihua, Sichuan	21	1400	101.45	26.45
Shimian (SM)	Yaan, Sichuan	27	1120	102.13	29.40
Jiuyuan (JY)	Wanyuan, Sichuan	15	900	108.21	31.84
Pengxian (PX)	Yaan, Sichuan	30	600	102.98	29.98
Muchuan (MC)	Muchuan, Sichuan	16	540	103.90	29.02
Wenchang (WC)	Wenchang, Hainan	20	10	110.87	19.72

Sampling occurred on local farms with owner permission. All procedures for sample collection were approved by the Institutional Animal Care and Use Committee of Sichuan Agricultural University under permit number DKY- S20163651.

### DNA amplification and sequencing

Primer pairs flanking the coding region of exons were designed by Primer Premier 6.0 [15]. The details for these primers are summarized in Tables 2 and 3. PCR was performed in 25 μL reactions that contained 50 ng DNA template, 1 × buffer (including 1500 μmol L^-1^Mg_2_Cl^2^, 200 μmol L^-1^ dNTPs, and 1.5 U of Taq DNA polymerase) and 1 μmol L^-1^ of each primer. Cycling parameters were as follows: initial denaturation at 96°C for 4 min, followed by 35 cycles of 95°C for 30 s, then annealing (temperatures provided in Tables 2 and 3) for 1 min, and 72°C for 90 s, and a final extension at 72°C for 10 min. PCR products were sequenced in both directions by the Beijing Genomics Institute (BGI).

**Table 2:** Primer information for detecting SNPs in PPARα coding regions.

Names	Target regions	Primer sequences (5’-3’)	Product length (bp)	Annealing temperature (°C)
P1	Exon1	F^1^: ACCTGTCAGAGATTCACATT	687	50.5
		R^2^: AAGGAGGCATTGATACTCAT		
P2	Exon2	F: GCTATGATTATCCACTACTGAC	702	58.4
		R: ATTGCCTCTGCTTGATGAA		
P3	Exon3	F: CTCAAGGCTCTCAGTTCTT	727	57.8
		R: GCAAGCAACCTACCAGAT		
P4	Exon4	F: TCATCAGTCAGGTCTCAGT	605	53.6
		R:CTACTATAACTTAGAGGCTCCT		
P5	Exon5	F: ACACGGCAGTTCACAGT	727	54.6
		R: CACCAACTCTCTTTACTTTCC		
P6	Exon6	F: TTAGTAGCACAGGTGGTATT	655	56.6
		R: AGCACTCCAGTTACTTAGC		

^1^ The forward sequence of the primer.

^2^ The reverse sequence of the primer.

**Table 3:** Primer information for detecting SNPs in *EGLN3* coding regions.

Names	Target regions	Primers sequence (5’-3’)	Production length (bp)	Annealing temperature (°C)
E1	Exon1	F^1^: CAAGATGCTGCGTGAAGTG	687	50.5
		R^2^: CTGGTATGAGGAAGGCGAAT		
E2	Exon2	F: AGGCTGTCACTAGATCACTA	702	58.4
		R: AGGCAGAGTCATCAACAAC		
E3	Exon3	F: CCAGTGTTGTCATATAGC	727	57.8
		R: ATCTGATGTTGGTAGGAG		
E4	Exon4	F: CCTCATCACCATCCTGTTC	605	53.6
		R:ACACCAGACTCATACTAAGAC		
E5	Exon5	F: GGAGCAGAAGGAGAACTATT	727	54.6
		R: CCAGCAACTTACTCTCAGAT		

^1^ The forward sequence of the primer.

^2^ The reverse sequence of the primer.

### Sequence data analysis

Sequence variations, including nucleotide composition and locations were identified by MEGA 5.10 [16]. The sequences were edited and aligned by DNAstar [17]. *PPARα* and *EGLN3* genome sequences of Chinese red jungle fowl that were obtained from NCBI GenBank (NC_ 006088.4 and NC_006092.4) were used as the reference sequences. Allele frequencies of *EGLN3* and *PPARα* genes in TC and LC groups were analyzed by Pearson’s Chi-square tests. These parameters were calculated using SPSS software Version 22 and *P* < 0.05 was considered significant. We used Arlequin 3.5 to calculate F_st_ and analyzed population genetic differentiation [18]. Phylogenetic analysis of nucleotide sequences was carried out by MEGA, J modeltest, BEAST2 and Figtree. J modeltest was used to estimate the best model for establishing the Phylogenetic tree and we used BEAST2 to construct the Phylogenetic tree. Figtree and MEGA were used to embellish the tree. The nucleotide sequences of *EGLN3* and *PPARα* in 8 representative vertebrates were used to construct the Phylogenetic tree and were retrieved from Ensembl [19].

### Protein secondary and tertiary structure prediction

Protein structure was predicted using SWISS-MODEL (https://www.swissmodel.expasy.org/). DNAstar was used for analyzing hydrophilicity. The complete genome of Cochin-Chinese Red Jungle Fowl was used as the reference sequence (ENSGALG00000041470).

## Results

### Sequence variations in *EGLN3* and *PPARα*

**Fig 2:**
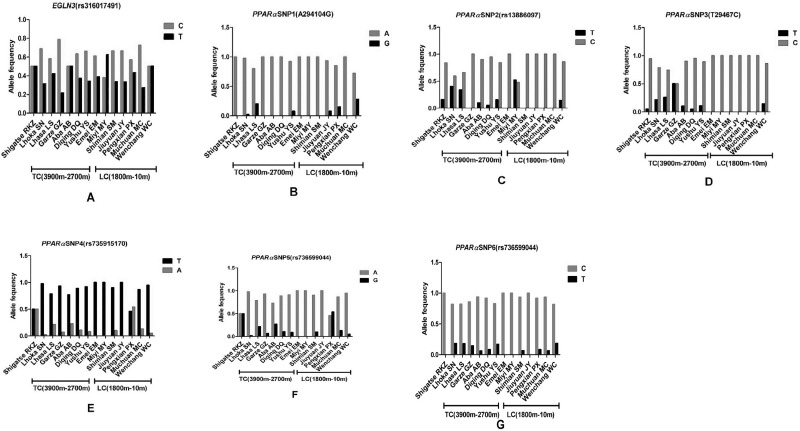
Allele frequencies of the SNPs scanned in genes *EGLN3* and *PPARα* for the populations at different altitude locations. (A) Pattern of allele frequencies at the SNP in *EGLN3*. “C” and “T” represent the frequencies of the ancestral and mutant alleles of the candidate SNP in each chicken population, respectively. (B)Pattern of allele frequencies at the SNP1 in *PPARα*. “A” and “G” represent the frequencies of the ancestral and mutant alleles of the candidate SNP in each chicken population, respectively. (C) Pattern of allele frequencies at the SNP2 in *PPARα*. “T” and “C” represent the frequencies of the ancestral and mutant alleles of the candidate SNP in each chicken population, respectively. (D)Pattern of allele frequencies at the SNP3 in *PPARα*. “T” and “C” represent the frequencies of the ancestral and mutant alleles of the candidate SNP in each chicken population, respectively. (E)Pattern of allele frequencies at the SNP4 in *PPARα*. “T” and “A” represent the frequencies of the ancestral and mutant alleles of the candidate SNP in each chicken population, respectively. (F)Pattern of allele frequencies at the SNP5 in *PPARα*. “A” and “G” represent the frequencies of the ancestral and mutant alleles of the candidate SNP in each chicken population, respectively. (G)Pattern of allele frequencies at the SNP6 in *PPARα*. “C” and “T” represent the frequencies of the ancestral and mutant alleles of the candidate SNP in each chicken population, respectively.

One SNP (rs316017491, C > T) was identified in *EGLN3*. Allele frequencies of *EGLN3* in TC and LC groups are shown in Table 4. The distribution of this SNP in each population is shown in Fig 2A and Table A in S1 File. The SNP is a synonymous substitution (Table 5). Pearson’s chi-square test results showed that there was no significant difference between TC and LC for allele distribution (*P* > 0.05).

**Table 4:** Allele frequencies of mutation loci in *EGLN3* and *PPARα* genes.

Genes	SNP	Allele distribution			*P*-Value^5^	MAF^6^
		Allele	TC^1^	LC^2^		
*EGLN3*	SNP rs316017491	C	183(0.614)^3^	143(0.572) ^3^	0.361	0.40511
		T	115(0.386)	107(0.428)		
*PPARα*	SNP1 (A29410^4^G)	A	243(0.929)	221(0.944)	0.596	0.06262
		G	18(0.071)	13(0.056)		
	SNP2 rs13886097	T	54(0.215)	24(0.103)	0.001**	0.16049
		C	198(0.785)	210(0.897)		
	SNP3 (T29467C)	T	42(0.166)	2(0.009)	0.000**	0.09053
		C	210(0.834)	232(0.991)		
	SNP4 rs735915170	T	267(0.862)	190(0.896)	0.293	0.12452
		A	43(0.138)	22(0.104)		
	SNP5 rs736599044	A	264(0.852)	190(0.890)	0.176	0.13027
		G	46(0.148)	22(0.104)		
	SNP6 rs740077421	C	267(0.861)	199(0.939)	0.008**	0.10727
		T	43(0.139)	13(0.061)		

^1^TC Tibetan chickens.

^2^LC Lowland chickens.

^3^The figures in brackets represent allele frequencies.

^4^ The number represents the SNP position in the DNA sequence.

^5^ Pearson’s Chi-square test.

^6^MAF represents the minor allele frequency.

** *P*-value less than 0.01.

**Table 5:** Mutation information for *EGLN3* and *PPARα*.

SNP locus	Genomic location	Nucleotide variant		Amino acid variant	
		Wild	Mutant	Wild	Mutant
*EGLN3*					
SNP rs316017491	Chr5:35744822	C	T	Phe	Phe
*PPARα*					
SNP1^1^ (A29410^2^G)		A	G	Arg	Arg
SNP2 rs13886097	Chr1:71358406	**T**	**C**	Ile	Met
SNP3 (T29467C)		T	C	Ile	Ile
SNP4 rs73591510	Chr1:71360891	T	A	Ser	Ser
SNP5 rs73659904	Chr1:71692243	A	G	Val	Val
SNP6 rs74007741	Chr1:71692264	C	T	Thr	Thr

^1^ SNP1 and SNP3 are unreported;

^2^ The number represents the SNP location in the nucleotide sequence.

Six SNPs were identified in *PPARα*. Their allele frequencies in groups TC and LC are shown in Table 4. This is the first report of SNP1 and SNP3. Distributions of six SNPs in each subgroup are shown in Fig 2B–2G and Tables B-G in S1 File. One non-synonymous mutation (rs13886097, T > C) and five synonymous mutations were found in *PPARα* (Table 5). All SNPs were observed in both TC and LC. That the minor allele frequency of all loci was greater than 1% suggests that mutation sites are ubiquitous. There were significant differences in allele frequencies between TC and LC for SNP2, SNP3, and SNP6 (*P* < 0.01), whereas there were no significant difference in allele frequencies between TC and LC for SNP1, SNP4, and SNP5 (*P* > 0.05).

Hardy-Weinberg equilibrium (HWE) test results showed that except for SNP1, SNP2, and SNP3 of *PPARα*, the other SNPs were consistent with HWE in TC groups (*P* > 0.05), whereas there was no SNP consistent with HWE (*P* < 0.01) in LC (Table 6). The observed heterozygosity of all SNPs was from 0.079 to 0.475 in TC and from 0 to 0.63 in LC.

**Table 6:** Hardy-Weinberg equilibrium (HWE) tests of SNPs in *EGLN3* and *PPARα* for Tibetan chickens and Lowland chickens.

Genes	SNP	Tibetan chickens				Lowland chickens			
		*χ*^*2*^	Pearson’s P	Ho^2^	He^3^	*χ*^*2*^	Pearson’s P	Ho	He
*EGLN3*	SNP rs316017491	0.004	0.950	0.47518	0.47401	10.514	0.001*	0.63	0.48963
*PPARα*	SNP1 (A29410G)	20.769	0.000*	0.07937	0.13192	42.488	0.000*	0.04274	0.10573
	SNP2 rs13886097	29.306	0.001*	0.1746	0.33755	NA^1^	NA	0	0.18478
	SNP3 (T29467C)	12.225	0.000*	0.190476	0.27689	NA	NA	0	0.01784
	SNP4 rs735915170	0.002	0.964	0.23871	0.23791	36.559	1.481E-09*	0.075472	0.18637
	SNP5 rs736599044	0.018	0.893	0.258065	0.2522	36.559	1.481E-09*	0.075472	0.19701
	SNP6 rs740077421	1.914	0.167	0.212903	0.23936	NA	NA	0.122642	0.11456

* represents *P*-value of less than 0.05.

^1^NA means not able to be calculated. (The value of *χ*^2^ is not calculated because the frequency of a certain genotype was 0 in the Lowland chickens)

^2^Ho represent the observed heterozygosity.

^3^ He represent the expected heterozygosity.

### Population genetic differentiation

Fix index statistic test (F_st_) values for each SNP locus of *EGLN3* and *PPARα* are displayed in Table 7. There was population differentiation between groups TC and LC for SNP2, SNP3, and SNP6 of *PPARα* (*P* < 0.05), while for other SNPs there were enough heterozygotes in the metapopulation (*P* > 0.05). Further analysis for SNP2, SNP3, and SNP6 indicated that the variation mainly occurred in the interior of the population (*P* < 0.05) and their values were 77.75%, 81.27%, and 96.18%, respectively (Table 8).

**Table 7:** F_st_ values for the SNPs in *EGLN3* and *PPARα* for Tibetan chickens and Lowland chickens.

Genes	SNP locus	F_st_ value	P-value
*EGLN3*	SNP rs316017491	0.001	0.286
*PPARα*	SNP1 (A29410G)	-0.002	0.603
	SNP2 rs13886097	0.041	0.000**
	SNP3 (T29467C)	0.138	0.000**
	SNP4 rs735915170	-0.001	0.407
	SNP5 rs736599044	0.005	0.160
	SNP6 rs740077421	0.025	0.006**

** represents *P*-value less than 0.01.

**Table 8:** Variance analysis of SNP2 in *PPARα* of TC and LC^1^.

SNP	Source of variation	Sum of squares	Variance components	Percentage variation	Fixation indices	P Value
SNP2	Among groups	1.514	-0.000	-0.001	-0.000	0.327
	Among populations within groups	13.365	0.031	22.251	0.222	0.000
	Within populations	50.602	0.107	77.749	0.222	0.000
	Total	65.481	0.138			
SNP3	Among groups	3.034	0.011	12.620	0.138	0.003
	Among populations within groups	2.994	0.005	6.106	0.070	0.003
	Within populations	33.989	0.072	81.274	0.187	0.000
	Total	40.016	0.089			
SNP6	Among groups	0.709	0.002	2.245	0.022	0.072
	Among populations within groups	1.764	0.002	1.571	0.016	0.069
	Within populations	47.587	0.093	96.182	0.038	0.018
	Total	50.061	0.097			

^1^ Tibetan chickens and Lowland chickens.

### Phylogenetic tree

**Fig 3:**
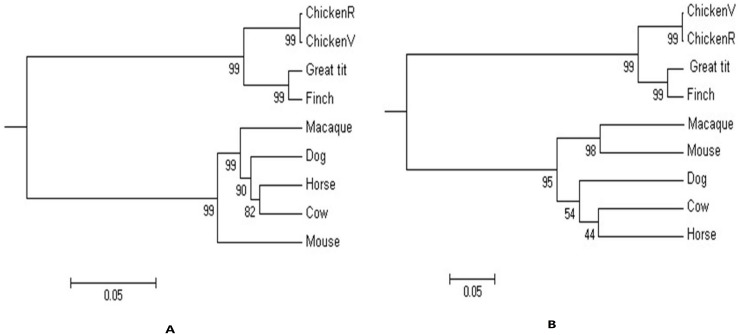
The phylogenetic tree of *EGLN3* and *PPARα*. (A) The phylogenetic tree of *EGLN3*. (B) The phylogenetic tree of *PPARα*. The gene IDs of the species in the Phylogenetic tree for *EGLN3* are as follows: Chicken, 423316; Finch, 100219039; Macaque, 101865926; Dog, 403654; Horse, 100056635; Cow, 535578; Mouse, 112407; Great Tit, 107206290. The gene IDs of the species in the Phylogenetic tree for *PPARα* are as follows: Chicken, 374120; Finch, 102037043; Macaque, 105489798; Dog, 480286; Horse, 100049840; Cow, 281992; Mouse, 19013; Great Tit, 107204346. R: represents the nucleotide sequence before mutation; V: represents the nucleotide sequence after mutation.

Nucleotide sequences of *EGLN3* from mouse, cow, horse, macaque, dog, chicken, great tit, and finch were used in phylogenetic analyses. Results showed that the phylogenetic tree was generally divided into two branches. One branch contains the chicken, great tit, and finch and another includes the macaque, dog, horse, cow, and mouse (Fig 3A). We found that the genetic distances of *EGLN3* among chicken, great tit, and finch were close.

The same analysis of *PPARα* was performed on mouse, cow, horse, macaque, dog, chicken, great tit, and finch. Similarly, the Phylogenetic tree was generally divided into two branches. Mouse, cow, horse, dog, and macaque formed a branch and the other species constituted another independent branch, with high homology among chicken, great tit, and finch (Fig 3B).

### Bioinformatics analysis of *PPARα*

**Fig 4:**
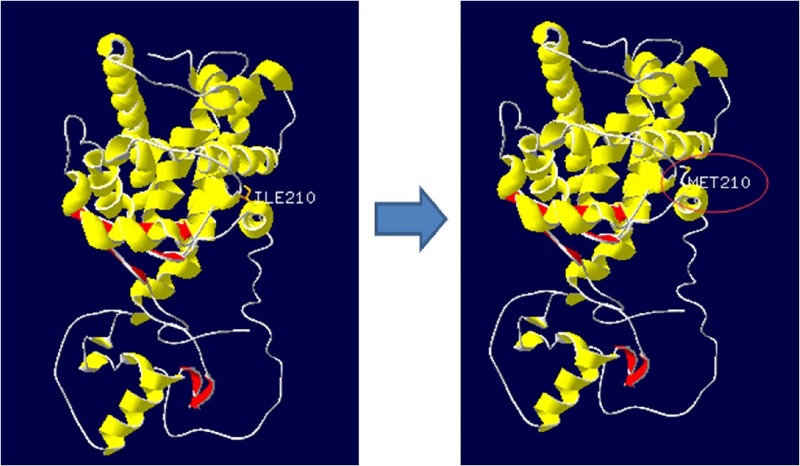
Three dimensional modeling of the amino acid sequence for *PPARα*. One non-synonymous mutation (Ile > Met) was identified.(A) The three-dimensional model before mutation. (B) The three-dimensional model after mutation. α-helix, β-strand, and random coil are represented with yellow, red, and grey, respectively.

**Fig 5:**
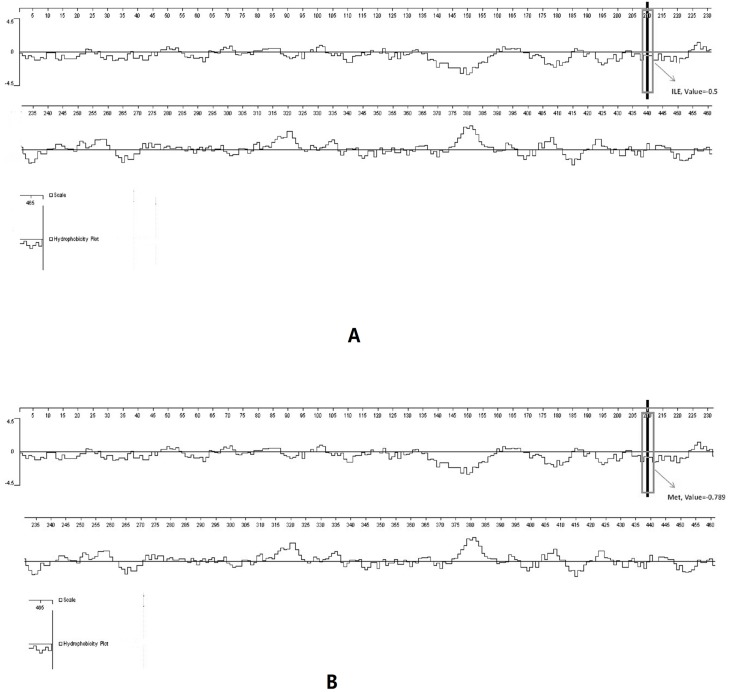
Protein hydrophobicity analyses for the PPARα protein. (A) Hydrophobic analysis before mutation; (B) Hydrophobic analysis after mutation; Positive values represent hydrophobic and negative values represent hydrophilic.

The SNP2 of *PPARα* resulted in an amino acid change (Ile > Met). The amino acid substitution occurred in the ligand-binding domain (LBD). Further study of this mutation site indicated that amino acid residues had changed (Fig 4) and that the mutated protein had higher hydrophilicity (Fig 5).

## Discussion

Chicken (*Gallus gallus*) is not only an important domestic bird for egg and meat production, but also a valuable model for evolutionary and developmental biology studies [20]. Tibetan chickens have inhabited the Tibetan plateau for thousands of years, and during that time have developed adaptability to hypoxia [21, 22]. Mutations in DNA and changes in functionality of proteins are responsible for these physiological adaptations to the hypoxic environment.

Herein, we analyzed the polymorphisms in *EGLN3* and *PPARα* genes in LC and TC populations. One and six SNPs were detected in *EGLN3* and *PPARα*, respectively. The MAF values for all SNPs were greater than 0.05, which is of great significance. For the *EGLN3* SNP, there was no significant difference between TC and LC in allele frequencies, whereas for SNP2, SNP3, and SNP6 in *PPARα*, there were significant differences between TC and LC in their respective allele frequencies. The mutant allele frequencies of SNP2 and SNP3 in LC were higher than those in Tibetan chickens and Hardy-Weinberg equilibrium (HWE) test results showed that all SNPs in LC were not consistent with HWE, indicating that the genetic structure of Lowland chickens may be affected by environmental or artificial factors[23]. The fixed index is a theoretical measure of whether the actual frequency of genotypes in a population departs from the genetic balance[24]. The result of fix index statistic tests showed there was a significant difference in the Fst value for SNP2, SNP3, and SNP6 between TC and LC, demonstrating that the three sites were specific in different populations and may be candidates for high altitude hypoxia adaptability. Arlequin was used to analyze the source of variation and the result showed that variation was mainly derived from individuals. These results suggest that geographic isolation among these groups diminished gradually, and likely did not play a major role in the genetic differentiation among populations [23].

Phylogenetic analyses showed that genetic relationships among chicken, great tit, and finch are close for *EGLN3* and *PPAR*α, which is consistent with the results of zoological classification [25]. This homology represents the proximity of species relationship, reflecting the importance of the structural stability of the *EGLN3* and *PPARα* gene among species.

Bioinformatics analyses indicated that except for SNP2 in *PPARα*, the other SNPs were synonymous mutations. Although synonymous mutations do not cause structural variation in the protein, it can change the amount of expression and modulate the translation efficiency of the downstream target protein [26].

In the present study, we identified one non-synonymous mutation at SNP2 (Ile > Met). The variation occurred in the ligand-binding domain (LBD), which contributes to the dimerization interface of the receptor and in addition, binds co-activator and co-repressor proteins [27].

The *PPARα* protein is highly hydrophobic [28], but the mutation detected in the present study increased its hydrophilicity and made it more likely to be hydrolyzed. As activated-*PPARα* modulates lipid metabolism by up-regulating the expression of key genes such as fatty acid transporter protein (FATP), carnitine palmitoyl transferase I (CPT I), and acetyl-CoA synthetase (ACS), we inferred that this genetic variation may alter the efficiency of lipid catabolism.

In conclusion, genetic analysis of *PPARα* and *EGLN3* genes in Tibetan and Lowland chickens suggests that the non-synonymous SNP2 of *PPARα* may play a role in the ability of Tibetan chickens to adapt to a high altitude environment.

## Supporting information

S1 File Allele and genotype frequencies of the SNPs in the PPARα and EGLN3 genes. Table A Allele and genotype frequencies of the SNP in the EGLN3 gene. Table B Allele and genotype frequencies of the SNP1 in the PPARα gene. Table C Allele and genotype frequencies of the SNP2 in the PPARα gene. Table D Allele and genotype frequencies of the SNP3 in PPARα gene. Table E Allele and genotype frequencies of the SNP4 in PPARα gene. Table F Allele and genotype frequencies of the SNP5 in the PPARα gene. Table G Allele and genotype frequencies of the SNP6 in the PPARα gene.
(DOCX)
