# Supplementary material for: Polymorphisms in the Egl nine homolog 3 (EGLN3) and Peroxisome proliferator activated receptor-alpha (PPARα) genes and their correlation with hypoxia adaptation in Tibetan chickens
Source: PLoS One. 2018 Mar 15;13(3):e0194156. doi: 10.1371/journal.pone.0194156 (PMC5854350; doi:10.1371/journal.pone.0194156)
Supplement: S1 File — Table A Allele and genotype frequencies of the SNP in the EGLN3 gene. Table B Allele and genotype frequencies of the SNP1 in the PPARα gene. Table C Allele and genotype frequencies of the SNP2 in the PPARα gene. Table D Allele and genotype frequencies of the SNP3 in PPARα gene. Table E Allele and genotype frequencies of the SNP4 in PPARα gene. Table F Allele and genotype frequencies of the SNP5 in the PPARα gene. Table G Allele and genotype frequencies of the SNP6 in the PPARα gene. (DOCX) [file pone.0194156.s001.docx]

**Supplementary Information**

**S1 File. Allele and genotype frequencies of the SNPs in the *PPARα* and *EGLN3* genes**

**Table A Allele and genotype frequencies of the SNP in the *EGLN3* gene**

| Genotype  /allele | Shigatse RKZ(10) | Lhoka SN(16) | Lhasa LS(25) | Garze GZ(7) | Aba AB(18) | Diqing DQ(15) | Yushu YS(50) | Emei EM(9) | Miyi MY(21) | Shimian SM(21) | Jiuyuan JY(15) | Pengxian PX(28) | Muchuan MC(11) | Wenchang WC(20) |
| --- | --- | --- | --- | --- | --- | --- | --- | --- | --- | --- | --- | --- | --- | --- |
| CC | 0.400  (4) | 0.438  (7) | 0.240  (6) | 0.571  (4) | 0.222  (4) | 0.400  (6) | 0.460  (23) | 0.333  (3) | 0.190  (4) | 0.380  (8) | 0.330  (5) | 0.214  (6) | 0.545  (6) | 0.000  (0) |
| CT | 0.200  (2) | 0.500  (8) | 0.680  (17) | 0.429  (3) | 0.556  (10) | 0.467  (7) | 0.400  (20) | 0.556  (5) | 0.380  (8) | 0.570  (12) | 0.670  (10) | 0.714  (20) | 0.364  (4) | 1.000  (20) |
| TT | 0.400  (4) | 0.063  (1) | 0.080  (2) | 0.000  (0) | 0.222  (4) | 0.133  (2) | 0.140  (7) | 0.111  (1) | 0.430  (9) | 0.050  (1) | 0.000  (0) | 0.071  (2) | 0.090  (1) | 0.000  (0) |
| C | 0.500 | 0.688 | 0.580 | 0.786 | 0.500 | 0.633 | 0.660 | 0.611 | 0.380 | 0.665 | 0.665 | 0.571 | 0.727 | 0.500 |
| T | 0.500 | 0.313 | 0.420 | 0.214 | 0.500 | 0.367 | 0.340 | 0.389 | 0.620 | 0.335 | 0.335 | 0.429 | 0.272 | 0.500 |

“C” represents the ancestral allele and “T” represents the mutant allele. Numbers represent allele/genotype frequencies, with the figures in brackets representing the number of individuals for each genotype.

**Table B Allele and genotype frequencies of the SNP1 in the *PPARα* gene**

| Genotype  /allele | | Shigatse RKZ(9) | | Lhoka SN(21) | | Lhasa  LS(25) | | Garze GZ(6) | Aba  AB(10) | Diqing DQ(10) | Yushu YS(45) | Emei EM(9) | Miyi MY(21) | Shimian SM(24) | Jiuyuan JY(15) | Pengxian PX(24) | Muchuan MC(17) | Wenchang WC(7) |
| --- | --- | --- | --- | --- | --- | --- | --- | --- | --- | --- | --- | --- | --- | --- | --- | --- | --- | --- |
| AA | 1.000  （9） | | 0.95  （20） | | 0.760（19） | | 1.000（6） | | 1.000（10） | 1.000  (10) | 0.844  (38) | 1.000  (9) | 1.000  (21) | 1.000  (24) | 0.867  (13) | 0.830  (20) | 1.000  (17) | 0.570  (4) |
| AG | 0.000  （0） | | 0.048  （1） | | 0.080（2） | | 0.0000（0） | | 0.000  (0) | 0.000  (0) | 0.156  (7) | 0.000  (0) | 0.000  (0) | 0.000  (0) | 0.133  (2) | 0.040  (1) | 0.000  (0) | 0.290  (2) |
| GG | 0.000  （0） | | 0.000  （0） | | 0.160（4） | | 0.000（0） | | 0.000  (0) | 0.000  (0) | 0.000  (0) | 0.000  (0) | 0.000  (0) | 0.000  (0) | 0.000  (0) | 0.130  (3) | 0.000  (0) | 0.140  (1) |
| A | 1.000 | | 0.976 | | 0.800 | | 1.000 | | 1.000 | 1.000 | 0.922 | 1.000 | 1.000 | 1.000 | 0.934 | 0.850 | 1.000 | 0.720 |
| G | 0.000 | | 0.024 | | 0.200 | | 0.000 | | 0.000 | 0.000 | 0.078 | 0.000 | 0.000 | 0.000 | 0.067 | 0.150 | 0.000 | 0.280 |

“A” represents the ancestral allele and “G” represents the mutant allele. Numbers represent allele/genotype frequencies, with the figures in brackets representing the number of individuals for each genotype.

**Table C Allele and genotype frequencies of the SNP2 in the *PPARα* gene**

| Genotype/allele | Shigatse RKZ(9) | Lhoka SN(21) | Lhasa LS(25) | Garze GZ(6) | Aba AB(10) | Diqing DQ(10) | Yushu YS(45) | Emei EM(9) | Miyi MY(21) | Shimian SM(24) | Jiuyuan JY(15) | Pengxian PX(24) | Muchuan MC(17) | Wenchang WC(7) |
| --- | --- | --- | --- | --- | --- | --- | --- | --- | --- | --- | --- | --- | --- | --- |
| TT | 0.000  (0) | 0.286  (6) | 0.240  (6) | 0.000  (0) | 0.100  (1) | 0.000  (0) | 0.067  (3) | 0.000  (0) | 0.524  (11) | 0.000  (0) | 0.000  (0) | 0.000  (0) | 0.000  (0) | 0.143  (1) |
| TC | 0.330  (3) | 0.238  (5) | 0.200  (5) | 0.000  (0) | 0.000  (0) | 0.100  (1) | 0.178  (8) | 0.000  (0) | 0.000  (0) | 0.000  (0) | 0.000  (0) | 0.000  (0) | 0.000  (0) | 0.000  (0) |
| CC | 0.670  (6) | 0.476  (10) | 0.560  (14) | 1.000  (6) | 0.900  (9) | 0.900  (9) | 0.755  (34) | 1.000  (9) | 0.476  (10) | 1.000  (24) | 1.000  (15) | 1.000  (24) | 1.000  (17) | 0.857  (6) |
| T | 0.160 | 0.405 | 0.340 | 0.000 | 0.100 | 0.050 | 0.156 | 0.000 | 0.524 | 0.000 | 0.000 | 0.000 | 0.000 | 0.143 |
| C | 0.840 | 0.595 | 0.660 | 1.000 | 0.900 | 0.950 | 0.844 | 1.000 | 0.476 | 1.000 | 1.000 | 1.000 | 1.000 | 0.857 |

“T” represents the ancestral allele and “C” represents the mutant allele. Numbers represent allele/genotype frequencies, with the figures in brackets representing the number of individuals for each genotype.

**Table D Allele and genotype frequencies of the SNP3 in *PPARα* gene**

| Genotype  /allele | Shigatse RKZ(9) | Lhoka SN(21) | Lhasa LS(25) | Garze GZ(6) | Aba AB(10) | Diqing DQ(10) | Yushu YS(45) | Emei EM(9) | Miyi MY(21) | Shimian SM(24) | Jiuyuan JY(15) | Pengxian PX(24) | Muchuan MC(17) | Wenchang WC(7) |
| --- | --- | --- | --- | --- | --- | --- | --- | --- | --- | --- | --- | --- | --- | --- |
| TT | 0.000  (0) | 0.095  (2) | 0.080  (2) | 0.500  (3) | 0.100  (1) | 0.000  (0) | 0.022  (1) | 0.000  (0) | 0.000  (0) | 0.000  (0) | 0.000 (0) | 0.000  (0) | 0.000  (0) | 0.143  (1) |
| TC | 0.111  (1) | 0.238  (5) | 0.360  (9) | 0.000  (0) | 0.000  (0) | 0.100  (1) | 0.178  (8) | 0.000  (0) | 0.000  (0) | 0.000  (0) | 0.000  (0) | 0.000  (0) | 0.000  (0) | 0.000  (0) |
| CC | 0.889  (8) | 0.667  (14) | 0.560  (14) | 0.500  (3) | 0.900  (9) | 0.900  (9) | 0.800  (36) | 1.000  (9) | 1.000  (21) | 1.000  (24) | 1.000  (15) | 1.000  (24) | 1.000  (17) | 0.857  (6) |
| T | 0.055 | 0.214 | 0.260 | 0.500 | 0.100 | 0.050 | 0.111 | 0.000 | 0.000 | 0.000 | 0.000 | 0.000 | 0.000 | 0.143 |
| C | 0.945 | 0.786 | 0.740 | 0.500 | 0.900 | 0.950 | 0.889 | 1.000 | 1.000 | 1.000 | 1.000 | 1.000 | 1.000 | 0.857 |

“T” represents the ancestral allele and “C” represents the mutant allele. Numbers represent allele/genotype frequencies, with the figures in brackets representing the number of individuals for each genotype.

**Table E Allele and genotype frequencies of the SNP4 in *PPARα* gene**

| Genotype  /allele | Shigatse RKZ(6) | Lhoka SN(22) | Lhasa LS(28) | Garze GZ(7) | Aba AB(24) | Diqing DQ(18) | Yushu YS(50) | Emei EM(9) | Miyi MY(21) | Shimian SM(15) | Jiuyuan JY(15) | Pengxian PX(12) | Muchuan MC(15) | Wenchang WC(19) |
| --- | --- | --- | --- | --- | --- | --- | --- | --- | --- | --- | --- | --- | --- | --- |
| TT | 0.000  (0) | 0.95  5(21) | 0.643  (18) | 0.857  (6) | 0.583  (14) | 0.778  (14) | 0.840  (42) | 1.000  (9) | 1.000  (21) | 0.800  (12) | 1.000  (15) | 0.417  (5) | 0.800  (12) | 0.895  (17) |
| TA | 1.000  (6) | 0.045  (1) | 0.286  (8) | 0.143  (1) | 0.375  (9) | 0.222  (4) | 0.160  (8) | 0.000  (0) | 0.000  (0) | 0.200  (3) | 0.000  (0) | 0.083  (1) | 0.133  (2) | 0.105  (2) |
| AA | 0.000  (0) | 0.000  (0) | 0.071  (2) | 0.000  (0) | 0.042  (1) | 0.000  (0) | 0.000  (0) | 0.000  (0) | 0.000  (0) | 0.000  (0) | 0.000  (0) | 0.500  (6) | 0.067  (1) | 0.000  (0) |
| T | 0.500 | 0.978 | 0.786 | 0.929 | 0.771 | 0.889 | 0.920 | 1.000 | 1.000 | 0.900 | 1.000 | 0.459 | 0.867 | 0.948 |
| A | 0.500 | 0.022 | 0.214 | 0.071 | 0.229 | 0.111 | 0.080 | 0.000 | 0.000 | 0.100 | 0.000 | 0.541 | 0.133 | 0.052 |

“T” represents the ancestral allele and “A” represents the mutant allele. Numbers represent allele/genotype frequencies, with the figures in brackets representing the number of individuals for each genotype.

**Table F Allele and genotype frequencies of the SNP5 in the *PPARα* gene**

| Genotype  /allele | Shigatse RKZ(6) | Lhoka SN(22) | Lhasa LS(28) | Garze GZ(7) | Aba  AB(24) | Diqing DQ(18) | Yushu YS(50) | Emei EM(9) | Miyi MY(21) | Shimian SM(15) | Jiuyuan JY(15) | Pengxian PX(12) | Muchuan MC(15) | Wenchang WC(19) |
| --- | --- | --- | --- | --- | --- | --- | --- | --- | --- | --- | --- | --- | --- | --- |
| AA | 0.000  (0) | 0.955  (21) | 0.643  (18) | 0.857  (6) | 0.500  (12) | 0.778  (14) | 0.820  (41) | 1.000  (9) | 1.000  (21) | 0.800  (12) | 1.000  (15) | 0.417  (5) | 0.800  (12) | 0.895  (17) |
| AG | 1.000  (6) | 0.045  (1) | 0.286  (8) | 0.143  (1) | 0.458  (11) | 0.222  (4) | 0.180  (9) | 0.000  (0) | 0.000  (0) | 0.200  (3) | 0.000  (0) | 0.083  (1) | 0.133  (2) | 0.105  (2) |
| GG | 0.000  (0) | 0.000  (0) | 0.071  (2) | 0.000  (0) | 0.042  (1) | 0.000  (0) | 0.000  (0) | 0.000  (0) | 0.000  (0) | 0.000  (0) | 0.000  (0) | 0.500  (6) | 0.067  (1) | 0.000  (0) |
| A | 0.500 | 0.978 | 0.786 | 0.929 | 0.729 | 0.889 | 0.910 | 1.000 | 1.000 | 0.900 | 1.000 | 0.459 | 0.867 | 0.948 |
| G | 0.500 | 0.022 | 0.214 | 0.072 | 0.271 | 0.111 | 0.090 | 0.000 | 0.000 | 0.100 | 0.000 | 0.541 | 0.133 | 0.052 |

“A” represents the ancestral allele and “G” represents the mutant allele. Numbers represent allele/genotype frequencies, with the figures in brackets representing the number of individuals for each genotype.

**Table G Allele and genotype frequencies of the SNP6 in the *PPARα* gene**

| Genotype/allele | Shigatse RKZ(6) | Lhoka SN(22) | Lhasa LS(28) | Garze GZ(7) | Aba AB(24) | Diqing DQ(18) | Yushu YS(50) | Emei EM(9) | Miyi MY(21) | Shimian SM(15) | Jiuyuan JY(15) | Pengxian PX(12) | Muchuan MC(15) | Wenchang WC(19) |
| --- | --- | --- | --- | --- | --- | --- | --- | --- | --- | --- | --- | --- | --- | --- |
| CC | 1.000  (6) | 0.727  (16) | 0.678  (19) | 0.714  (5) | 0.875  (21) | 0.889  (16) | 0.680  (34) | 1.000  (9) | 1.000  (21) | 0.867  (13) | 1.000  (15) | 0.833  (10) | 0.867  (13) | 0.632  (12) |
| CT | 0.000  (0) | 0.182  (4) | 0.286  (8) | 0.286  (2) | 0.125  (3) | 0.056  (1) | 0.300  (15) | 0.000  (0) | 0.000  (0) | 0.133  (2) | 0.000  (0) | 0.167  (2) | 0.133  (2) | 0.368  (7) |
| TT | 0.000  (0) | 0.091  (2) | 0.036  (1) | 0.000  (0) | 0.000  (0) | 0.055  (1) | 0.020  (1) | 0.000  (0) | 0.000  (0) | 0.000  (0) | 0.000  (0) | 0.000  (0) | 0.000  (0) | 0.000  (0) |
| C | 1.000 | 0.818 | 0.821 | 0.857 | 0.938 | 0.917 | 0.83 | 1.000 | 1.000 | 0.934 | 1.000 | 0.917 | 0.934 | 0.816 |
| T | 0.000 | 0.182 | 0.179 | 0.143 | 0.062 | 0.083 | 0.17 | 0.000 | 0.000 | 0.066 | 0.000 | 0.083 | 0.066 | 0.184 |

“C” represents the ancestral allele and “T” represents the mutant allele. Numbers represent allele/genotype frequencies, with the figures in brackets representing the number of individuals for each genotype.
